# Dysthyroid Optic Neuropathy: Treatment with Additional Intravenous Methylprednisolone Pulses after the Basic Schedule Is Associated with Stabilization or Further Improvement of Clinical Outcome

**DOI:** 10.3390/jcm11082068

**Published:** 2022-04-07

**Authors:** Maryla Pelewicz, Joanna Rymuza, Katarzyna Pelewicz, Piotr Miśkiewicz

**Affiliations:** Department of Internal Medicine and Endocrinology, Medical University of Warsaw, 02-091 Warsaw, Poland; mpelewicz@gmail.com (M.P.); joanna.paplinska@wp.pl (J.R.); katarzyna.pelewicz@gmail.com (K.P.)

**Keywords:** Graves’ orbitopathy, Graves’ ophthalmopathy, dysthyroid optic neuropathy, visual acuity, methylprednisolone, glucocorticoids

## Abstract

Background: Dysthyroid optic neuropathy (DON) is a sight-threatening complication of Graves’ orbitopathy (GO). Treatment of DON consists of the urgent administration of intravenous methylprednisolone (ivMP) in very high doses followed by orbital decompression if the response is poor or absent. It is advised to continue the therapy with pulses of ivMP in a weekly schedule. The purpose of this study was to evaluate the impact of the additional treatment with ivMP in a 12-week protocol on visual acuity (VA), color vision, clinical activity score (CAS) and proptosis in patients with DON. Methods: This study was performed on 19 patients with DON (26 eyes) treated with ivMP in very high doses, with further orbital decompression in 11 individuals (15 eyes). VA, color vision, CAS and proptosis were evaluated prior to the DON treatment, before and after the 12-week ivMP (first and last pulse). Additionally follow up was performed (22 eyes). Results: VA and color vision improved between the first and last pulse of the additional ivMP treatment (*p* = 0.04 and *p* = 0.003, respectively). CAS and proptosis were reduced at the end of the 12-week ivMP therapy compared to observations at the beginning (*p* < 0.001 and *p* = 0.04, respectively). Follow up confirmed stabilization of this achievement. Conclusions: The results of this study suggest that additional treatment with 12 pulses of ivMP improves or stabilizes the outcome of basic therapy in patients with DON.

## 1. Introduction

Graves’ orbitopathy (GO) is the most frequent extrathyroidal manifestation of Graves’ disease [1]. This autoimmune orbital disorder is characterized by pain, diplopia, disfiguring proptosis, swelling and redness of the eyelids [2,3]. The pathogenesis involves expansion of the orbital connective tissue and enlargement of the eye muscles caused by inflammation, adipogenesis and overproduction of glycosaminoglycans [4,5]. Dysthyroid optic neuropathy (DON) occurs in approximately 5% of patients with GO, and may lead to permanent loss of vision [6,7]. This severe complication results from optic nerve compression caused by swollen muscles and fat in the orbital apex [7,8]. Patients with DON may suffer from poor visual acuity (VA), impaired color vision and restricted visual field [4,6]. Diagnosis of DON is based on clinical, ophthalmological and radiological evaluation. Ophthalmological examination includes assessment of VA, color vision, visual fields, visual evoked potentials and optic fundus [9]. Radiological evaluation in the diagnosis of DON can be performed with magnetic resonance imaging (MRI) or computed tomography. It is used to assess apical optic nerve compression (apical crowding) and optic nerve stretching, and to exclude other orbital pathologies. In patients with insufficient response to the recommended basic treatment, radiological reassessment should be considered to establish further therapeutic measures [8,10,11].

It has been observed that a significant improvement in VA can be achieved by using very high doses of intravenous methylprednisolone (ivMP) or combination therapy with orbital decompression [12,13,14]. The 2021 European Group on Graves’ Orbitopathy (EUGOGO) guidelines recommend ivMP pulse therapy (0.5–1 g for three consecutive days or on every second day) as the first-line treatment of DON, which may be repeated for another week. In case of poor or absent response, surgical decompression needs to be performed within 2 weeks. If DON has resolved or improved, it is suggested to include additional pulses of ivMP (0.5 g) in a weekly schedule as a further step of the therapeutic process [11].

Previous EUGOGO guidelines recommended continuing ivMP treatment in a 12-week protocol (6 × 0.5 g and 6 × 0.25 g) when the basic treatment with very high doses of ivMP with/without decompression was completed [15,16]. Nevertheless, clear criteria of resolution of DON do not exist. The end point of basic therapy has not been specified. Improvement of VA is a crucial decision factor. However, some signs of DON often persist after completion of basic therapy (clinical: decreased visual acuity, visual field defects, reduced color vision; ophthalmic: swollen or pale optic disk; radiological: apical crowding). DON is a sight-threatening disease. Optimal therapy should be performed as early as possible because delay causes permanent changes in the optic nerve. The relapse or worsening of one or more signs of impaired optic nerve function after basic therapy were described in the previous studies [17,18].

Positive impact of the 12-week ivMP protocol on eye symptoms has been proven in moderate-to-severe and active GO [19]. So far, the influence of the additional 12-pulse ivMP therapy on DON patients was analyzed regarding only quality of life [20]. Therefore, we do not possess any data which verifies the purpose of the additional therapy with 12 pulses of ivMP or its effect on other features of DON once basic therapy has been completed.

The aim of this study was to evaluate the impact of the additional treatment with ivMP in a 12-week protocol on VA, color vision, clinical activity score (CAS) and proptosis in patients with DON.

## 2. Materials and Methods

### 2.1. Patients

Nineteen individuals diagnosed with DON were consecutively recruited from the Department of Internal Medicine and Endocrinology, Medical University of Warsaw, from 2011 to 2019. A total number of 26 eyes were affected with DON. The diagnosis of DON was based on at least two signs, including deterioration of VA (<1.0), loss of color vision (more than two errors in Ishihara plates), optic disc swelling and signs of DON in MRI (i.e., presence of apical crowding, optic nerve stretching) [14]. The inclusion criterion was additional therapy with ivMP in a 12-week protocol after the treatment of DON. Patients who received at least 6 pulses were included. The exclusion criteria were other diseases affecting visual function, such as cataract, glaucoma, high myopia and corneal exposition. 

### 2.2. Treatment

Treatment of DON was conducted as described in the EUGOGO guidelines [15,16]. Each individual received ivMP pulses in very high doses (0.5 g or 1 g for 3 consecutive days). Due to poor improvement, 9 individuals were administered two or more cycles of ivMP pulses (3 × 0.5 g or 3 × 1 g). Orbital decompression was performed in 11 individuals, in a total number of 15 eyes with DON. Afterwards, all patients were qualified for treatment with additional ivMP pulses in a 12-week protocol. A cumulative dose of 4.5 g (6 × 0.5 g and 6 × 0.25 g) was given to 18 individuals. One patient was qualified for 12 pulses of ivMP with a total dose of 7.5 g (6 × 0.75 g and 6 × 0.5 g), but due to the increased level of aspartate and alanine aminotransferases (AST and ALT, respectively) received 9 pulses of ivMP (cumulative dose 6 g).

### 2.3. Laboratory and Ophthalmological Evaluation

The serum levels of free triiodothyronine (fT3), free thyroxine (fT4), thyroid-stimulating hormone (TSH) and thyrotropin receptor antibodies (TSHR-Ab) were measured with an electro-chemiluminescent immunoassay performed on a Cobas 6000 analyzer from Roche Diagnostics (Mannheim, Germany). The reference ranges were as follows: TSH 0.27–4.2 μIU/mL; fT3 3.1–6.8 pmol/L; fT4 12–22 pmol/L; TSHR-Ab < 1.75 IU/L. VA was verified using Snellen charts and converted to logarithm of the minimum angle of resolution (logMAR) for statistical analysis. Color vision was tested using Ishihara plates. CAS was estimated for each eye based on two symptoms (orbital ache and gaze-evoked pain) and five signs (eyelid erythema or oedema, conjunctival redness, chemosis and swelling of the plica or caruncle). Active GO was diagnosed if CAS was ≥3/7 [11]. We used the Gorman score to evaluate and classify diplopia on a 4-point scale: 0, no diplopia; 1, intermittent diplopia; 2, inconstant diplopia; 3, constant diplopia [21]. Due to monocular vision in one of the patients, assessment of diplopia was performed in 18 individuals. Proptosis was measured in millimeters with a Hertel exophthalmometer at the same intercanthal distance for each patient. All parameters were evaluated at three time points: prior to the administration of ivMP as the first-line treatment for DON, before (first pulse) and after (last pulse) the additional ivMP treatment in a 12-week protocol.

After the therapy, a follow-up evaluation of the patients was made including assessment of VA, color vision, CAS, proptosis and TSHR-Ab.

The serum levels of glucose (GLU), AST, ALT, triglycerides (TG), total cholesterol (TC), low-density lipoprotein cholesterol (LDLC) and high-density lipoprotein cholesterol (HDLC) were measured after a 10 h fasting period (Cobas 8000 System, Roche Diagnostics, Switzerland). The reference ranges were as follows: GLU < 100 mg/dL, AST 7–56 IU/L, ALT 7–56 IU/L, TG 50–150 mg/dL, TC 120–200 mg/dL, LDLC < 130 mg/dL, HDLC > 40 mg/dL. Body mass index (BMI) was calculated using the formula: BMI = weight (kg)/height (m^2^). Mean arterial pressure (MAP) was measured based on systolic and diastolic blood pressure (SBP and DBP, respectively) with the following formula: MAP = 1/3 SBP + 2/3 DBP. Each parameter was evaluated at the beginning and after the additional treatment with 12 pulses of ivMP.

### 2.4. Statistical Analysis

All analyses were performed using SPSS statistical software version 22.0 (IBM SPPS Statistics, New York, NY, USA). Continuous variables are expressed as median values with interquartile range (25th–75th percentile). The Shapiro–Wilk test was used to confirm or reject the normal distribution of each continuous variable. Comparisons between continuous data were performed using Wilcoxon rank sum test. Statistical significance was established for results with a two-tailed *p*-value < 0.05.

## 3. Results

Demographic details as well as clinical and laboratory characteristics are presented in Table 1 and Table 2, respectively. The diagnosis of DON was made in 19 patients in a total number of 26 eyes (12 patients with unilateral DON and 7 patients with bilateral DON). Patients received a median cumulative dose of 7.5 (7.5–10.5) g ivMP.

**Figure 1:**
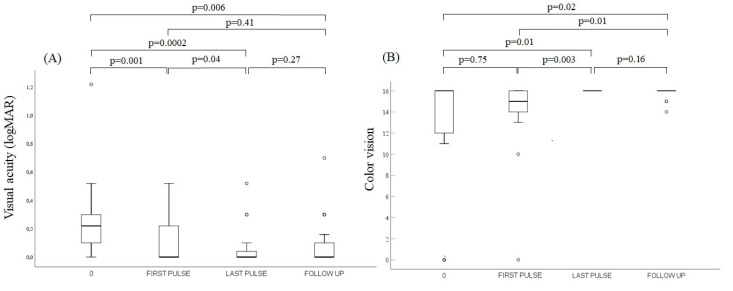
Comparison of variables during the treatment at three time points: 0, FIRST PULSE and LAST PULSE (*n* = 26) and after the treatment at FOLLOW UP (*n* = 22). (**A**) Visual acuity (logMAR). (**B**) Color vision. Vertical line ranges from maximum to minimum value. Data are shown as median values (line across the box) with interquartile (25th–75th percentile) range (the box). Bullets represent the outliers. Abbreviations: logMAR, logarithm of the minimum angle of resolution; 0, diagnosis of dysthyroid optic neuropathy; FIRST PULSE and LAST PULSE, before and after the additional treatment with intravenous methylprednisolone pulses, respectively; FOLLOW UP, post treatment evaluation after median time of 4.5 months.

**Figure 2:**
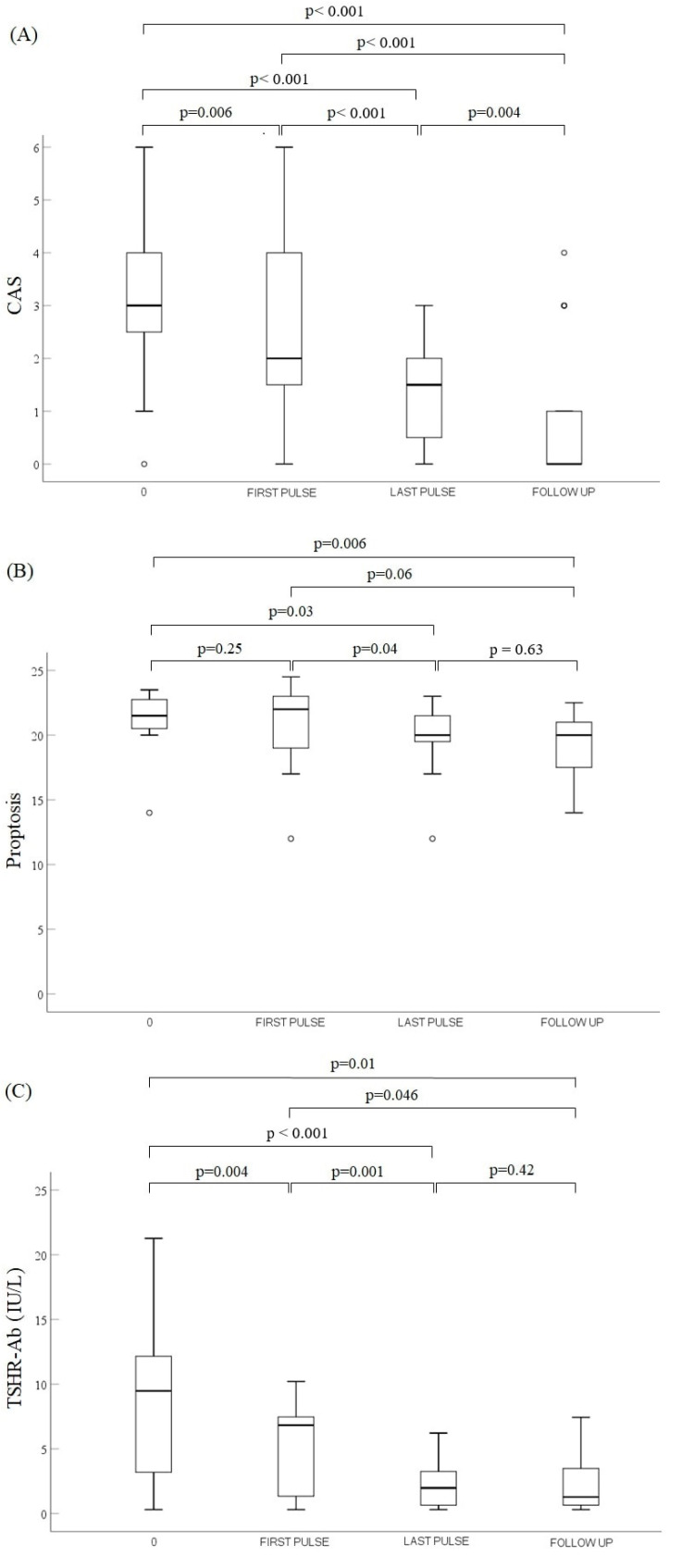
Comparison of variables during the treatment at three time points: 0, FIRST PULSE and LAST PULSE (*n* = 26) and after the treatment at FOLLOW UP (*n* = 22): (**A**) CAS, (**B**) proptosis and (**C**) TSHR-Ab. Vertical line ranges from maximum to minimum value. Data are shown as median values (line across the box) with interquartile (25th–75th percentile) range (the box). Bullets represent the outliers. Abbreviations: CAS, clinical activity score; TSHR-Ab, thyrotropin receptor antibodies; 0, diagnosis of dysthyroid optic neuropathy; FIRST PULSE and LAST PULSE, before and after the additional treatment with intravenous methylprednisolone pulses, respectively; FOLLOW UP, post-treatment evaluation after median time of 4.5 months.

VA and color vision significantly improved between the first and last pulse of the ivMP treatment in a 12-week protocol (*p* = 0.04 and *p* = 0.003, respectively). CAS, proptosis and TSHR-Ab reduced remarkably after the last pulse of the 12-week ivMP protocol compared to the first pulse (*p* < 0.001, *p* = 0.04 and *p* = 0.001, respectively). Median values of VA and color vision are shown in Figure 1. Median values of CAS, proptosis and TSHR-Ab are presented in Figure 2.

Assessment of double vision was constricted due to the orbital decompression that was performed in 11 patients; we observed deterioration of double vision in 5 of them. Increased levels of AST and ALT (49 IU/L and 67 IU/L) were observed in one of the patients after the ninth ivMP pulse. No significant changes in the serum levels of GLU, AST, ALT, TG, TC, LDLC and HDLC, nor in BMI and MAP, were found between the first and the last pulse of the additional ivMP treatment. Median values are presented in Table 3.

Follow up was performed after the median time of 4.5 (3–10) months from the last ivMP pulse. Two patients (four eyes) were lost to follow up. VA and color vision remained stable comparing to the assessment after the last pulse in all 17 patients (22 eyes) available for evaluation (*p* = 0.27 and *p* = 0.16, respectively). In the additional analysis between the first pulse of the additional treatment and the follow up, stabilization of VA and improvement of color vision (*p* = 0.41 and *p* = 0.01, respectively) were stated in the patients available for the reassessment (22 eyes). Median values of VA and color vision at follow up are shown in Figure 1, while those for CAS, proptosis and TSHR-Ab are shown in Figure 2.

## 4. Discussion

According to the 2021 EUGOGO guidelines, the management of DON should involve the immediate administration of ivMP in very high doses followed by urgent orbital decompression in case of poor or absent response within 2 weeks. Furthermore, if DON has resolved or improved it is advised to implement additional pulses of ivMP (0.5 g) in every week schedule with cumulative dose < 8 g [11]. However, no study has investigated the benefits of this additional therapy, whether its main goal is to maintain the results of basic DON treatment or to accomplish further improvement.

Our study showed an improvement of VA and color vision after the additional ivMP therapy compared to at its beginning. Moreover, significant reductions in CAS, proptosis and TSHR-Ab were achieved. The positive effect of the additional ivMP therapy was observed in overall assessment including follow up (four eyes were lost to the analysis): stabilization of VA and improvement of color vision were stated. Further ivMP therapy was not required. The results of our study indicate that patients with DON may benefit from additional treatment with ivMP after basic DON therapy.

Furthermore, we observed an increase of VA obtained after the basic therapy of DON (very high doses of ivMP with or without orbital decompression), which confirms the validity and efficacy of this treatment [12,13].

According to the current EUGOGO guidelines, due to the higher rate of adverse effects, cumulative doses of ivMP in GO treatment should not exceed 8.0 g per cycle, with the exception of DON treatment [11]. Previous research proved that therapy of GO with higher doses (1.0 g) of ivMP in consecutive days is associated with higher risk of liver dysfunction than treatment with moderate doses (≤0.5 g) in a 12-week protocol [22]. Another report showed that high doses of ivMP in a 12-week protocol may be considered relatively safe [23]. In our study, the cumulative dose of ivMP during the basic and additional therapy altogether exceeded 8 g in nine patients. Adverse effects (mild increase of transaminases) were experienced only by one of the patients. Due to the risk of permanent deterioration or loss of vision, DON must be treated aggressively with high ivMP doses. Our study proved the effectiveness and the lack of serious side effects of the additional therapy in patients with DON, and therefore it should be applied in the management of DON.

To our knowledge, this is the first study evaluating the impact of the additional treatment with 12 pulses of ivMP on VA, color vision, CAS and proptosis in patients with DON. The main limitations of our study are its retrospective character and the small number of patients. However, the majority of the reports regarding DON have a small sample size due to the low prevalence of the disease. Moreover, delayed effects of basic DON treatment, especially after decompression, should be considered. The study would also have benefited from a randomized design: one group treated with additional ivMP therapy according to the 2021 EUGOGO guidelines, and the other without additional therapy.

## 5. Conclusions

Inclusion of additional treatment with 12 pulses of ivMP into the combined therapy of DON provided positive results in the study participants. Results suggest that once the basic treatment of DON is over (pulses with ivMP in very high doses with or without orbital decompression), stabilization or further improvement in clinical features of DON (e.g., VA, color vision) may be achieved. Moreover, clinical characteristics of GO, including CAS and proptosis, could be reduced significantly. Additional therapy may prevent the relapse of DON.

This study indicates that additional treatment with 12 pulses of ivMP improves or stabilizes the outcome of basic therapy in patients with DON.

## Figures and Tables

**Table 1:** Baseline demographic characteristics.

Age, years	65 (50–72)
Male/female	6/13
Number of eyes with DON	26
Number of eyes with DON after decompression	15
Duration of GO, months	7 (4–31)
Duration of DON treatment, months	4 (4–6)
**Thyroid treatment before DON ^a^**	
Levothyroxine	1 (5.3)
Antithyroid drugs	8 (42.1)
Antithyroid drugs with levothyroxine	1 (5.3)
Radioiodine then levothyroxine	6 (31.6)
Thyroidectomy then levothyroxine	2 (10.5)
None	1 (5.3)
**Thyroid treatment during the additional therapy ^a,b^**	
Levothyroxine	8 (42.1)
Antithyroid drugs	3 (15.8)
Antithyroid drugs with levothyroxine	7 (36.8)
None	1 (5.3)

Continuous variables are presented as median (interquartile range) or number (percentage). ^a^ Number of patients. ^b^ Additional therapy of DON with 12 pulses of intravenous methylprednisolone. Abbreviations: DON, dysthyroid optic neuropathy; GO, Graves’ orbitopathy.

**Table 2:** Laboratory and clinical characteristics at three time points.

	0	FIRST PULSE	LAST PULSE
**Laboratory characteristics of thyroid disease**			
TSH, µIU/mL	0.69 (0.25–2.25)	0.39 (0.02–1.25)	1.07 (0.46–1.6)
fT3, pmol/L	4.64 (4.24–7.0)	4.75 (4.08–7.16)	4.42 (3.99–4.77)
fT4, pmol/L	18.4 (16.0–20.0)	16.41 (15.1–20.7)	16.23 (14.36–18.07)
TSHR-Ab, IU/L	11.85 (2.39–14.02)	6.49 (0.95–7.71)	2.09 (0.71–3.89)
**Clinical characteristics of orbital disease**			
Gorman score ^a^			
No diplopia	6 (33.3)	5 (27.8)	4 (22.2)
Intermittent diplopia	1 (5.6)	3 (16.7)	1 (5.6)
Inconstant diplopia	8 (44.4)	2 (11.1)	4 (22.2)
Constant diplopia	3 (16.7)	8 (44.4)	9 (50)
Visual acuity (logMAR)	0.22 (0.04–0.3)	0 (0–0.175)	0 (0–0.04)
CAS ^b^	3.0 (3.0–4.0)	2.0 (1.0–3.0)	1.0 (0.0–2.0)
Proptosis	21.0 (19.5–22.3)	20.25 (18.0–23.0)	19.5 (18.0–2-.5)
Eyes with active GO	27 (93.1)	15 (51.7)	4 (13.8)

Continuous variables are presented as median (interquartile range) or number (percentage). ^a^ Number of patients. ^b^ Activity was diagnosed if CAS was ≥3/7. Abbreviations: CAS, clinical activity score; GO, Graves’ orbitopathy; logMAR, logarithm of the minimum angle of resolution; TSH, thyroid-stimulating hormone; fT3, free triiodothyronine; fT4, free thyroxine; TSHR-Ab, thyrotropin receptor antibody; 0, diagnosis of dysthyroid optic neuropathy; FIRST PULSE and LAST PULSE, before and after the additional treatment with intravenous methylprednisolone pulses, respectively.

**Table 3:** Laboratory characteristics of patients with dysthyroid optic neuropathy before and after additional treatment with pulses of intravenous methylprednisolone.

	FIRST PULSE	LAST PULSE	*p*
Glucose, mg/dL	88 (85–101)	94 (86–99)	0.73
Lipid profile			
Triglycerides, mg/dL	88 (82–97)	98 (85–131)	0.94
Total cholesterol, mg/dL	196 (180–242)	212 (166–235)	0.78
Low-density lipoprotein cholesterol, mg/dL	119 (104–136)	109 (84–151)	0.87
High-density lipoprotein cholesterol, mg/dL	62 (54–65)	65 (62–68)	0.36
Liver function			
Aspartate aminotransferase, IU/L	19 (16–21)	17.5 (15–23)	0.69
Alanine aminotransferase, IU/L	21 (13–25)	16 (15–24)	0.51
Body mass index, kg/m^2 a^	24.5 (24.1–27.8)	25 (24–27.6)	0.92
Mean arterial pressure, mmHg ^b^	86 (83–92)	90 (92–97)	0.31

Data are shown as median (interquartile range). ^a^ BMI was calculated using the formula: BMI = weight (kg)/height (m^2^). ^b^ MAP was established with the following formula: MAP = 1/3 SBP + 2/3 DBP. Abbreviations: BMI, body mass index; MAP, mean arterial pressure; SBP, systolic blood pressure; DBP, diastolic blood pressure; FIRST PULSE and LAST PULSE, before and after the additional treatment with pulses of intravenous methylprednisolone, respectively.

## Data Availability

The data of this study are available on request from the corresponding author.
